# The Apple Vision Pro as a Neurosurgical Planning Tool: A Case Report

**DOI:** 10.7759/cureus.54205

**Published:** 2024-02-14

**Authors:** Joshua Olexa, Annie Trang, Jonathan Cohen, Kevin Kim, Maureen Rakovec, Jordan Saadon, Charles Sansur, Graeme Woodworth, Gary Schwartzbauer, Jacob Cherian

**Affiliations:** 1 Neurosurgery, University of Maryland School of Medicine, Baltimore, USA; 2 Neurosurgery, Hoth Intelligence, Philadelphia, USA

**Keywords:** neurosurgery, vision pro, apple, augmented reality, mixed reality, apple vision pro

## Abstract

With its recent release, the Apple Vision Pro (Apple Inc., Cupertino, CA) represents a promising technological advancement of mixed reality in the field of neurosurgery and medicine more broadly. With all new technologies, it is critical to facilitate early use and assessment of the technology to facilitate adoption by the larger medical community. A 44-year-old female with a history of ruptured intracranial aneurysm status post anterior communicating artery aneurysm clipping presented with worsened confusion and intermittent headache. CT imaging revealed evidence of hydrocephalus due to the malfunction of a previous right parietal ventriculoperitoneal (VP) shunt. Prior to the case, the Apple Vision Pro was used in the operating room to visualize and interact with a 3D model of the patient's anatomy for the patient undergoing a VP shunt placement. A visualization of the 3D model through the headset was used to plan the approach and entry point. At the conclusion of the procedure, all clinicians and operating staff who used the technology for planning completed a survey about their initial impressions of the headset. Overall, users felt the 3D models felt realistic (4.5/5), that the display of the user's real-world view felt natural (4.3/5), and that the headset did not cause eye strain or fatigue (4.5/5). The majority of users responded that they would continue to use the headset for cases (4/5). This represents one of the first known clinical uses of the Apple Vision Pro. It is a cutting-edge technology that will likely provide immense value for healthcare providers as it becomes more integrated into clinical care.

## Introduction

In a landmark release, Apple unveiled the latest marvel in spatial computing technology, the Apple Vision Pro (Apple Inc., Cupertino, CA). This advancement pushes traditional boundaries, ushering in a new era of possibilities for the field of medicine. With its state-of-the-art features and groundbreaking applications, the Apple Vision Pro is set to redefine the landscape of healthcare, offering unprecedented tools for medical professionals and promising transformative impacts on patient care [1-2]. In the field of medical mixed reality, neurosurgeons in particular have been leaders in pushing this class of technologies and driving the integration of mixed reality into clinical care [3-6].

In the field of neurosurgery, the pursuit of precision and innovation is paramount. Recognizing the advantages as well as disadvantages of previous augmented reality (AR) systems, Apple Vision Pro may represent a cutting-edge technology that can elevate neurosurgical procedures to greater levels of precision and efficiency [7-8]. The system is a next-generation mixed reality device with twelve cameras built into it that enable high-resolution scene understanding and eye tracking. Additionally, various sensors, including light detection and ranging scanners, depth sensors, and inertial measurement units, allow for high accuracy and low-latency spatial computing. All of these components provide a user with a seamless experience of displaying digital information in their surrounding real-world view [7-8]. Given the novelty of the system, we set out to evaluate the new headset for the surgical planning of a ventriculoperitoneal (VP) shunt placement case. Even though the device is in its early stages, this visionary technology is poised to have a tremendous impact on the field of neurosurgery and medicine more generally. The release of this device has been anticipated for years. As such, this report represents a major leap forward in the field of mixed reality, as it is one of the earliest known uses of this technology in clinical practice.

## Case presentation

**Figure 1:**
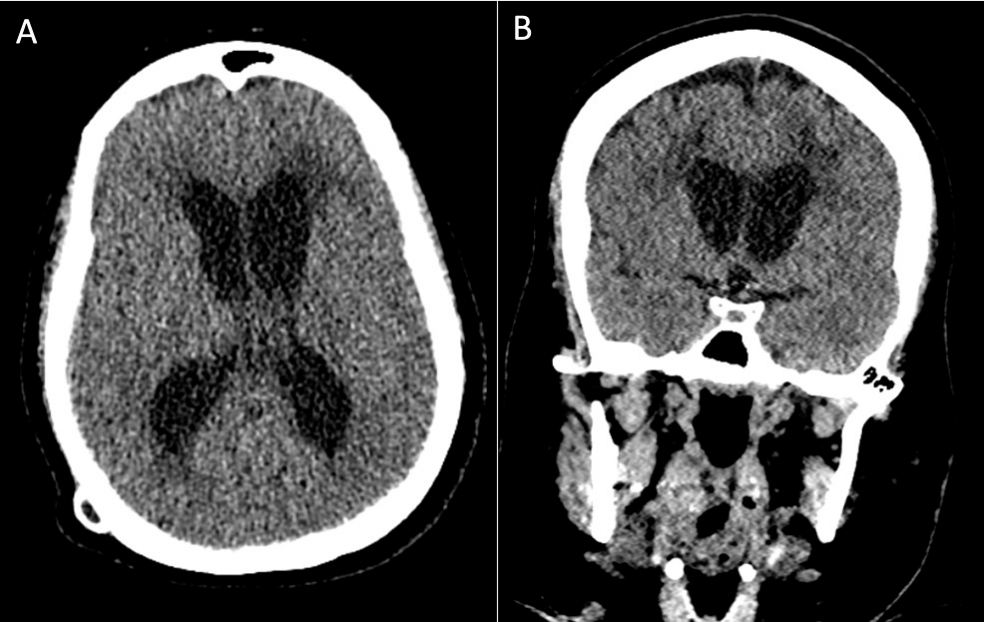
Preoperative CT imaging (A) axial and (B) coronal preoperative computed tomography (CT) scans

A 44-year-old female with a history of ruptured intracranial aneurysm status post anterior communicating artery aneurysm clipping presented with worsened confusion and intermittent headache. CT imaging revealed evidence of hydrocephalus due to the malfunction of a previous right parietal VP shunt (Figure 1).

3D model generation

A 3D model of the patient’s anatomy was generated from digital imaging and communications in medicine imaging using autosegmentation algorithms. The brain (orange), skull (white), face (gray), and ventricles (blue) were included in the 3D model.

Apple Vision Pro and software

**Figure 2:**
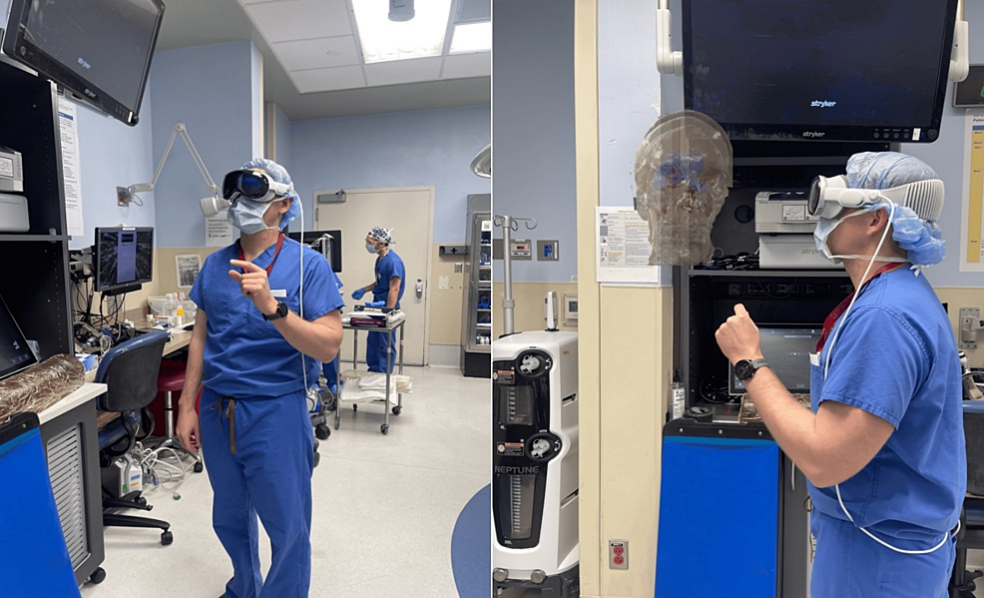
Neurosurgeon wearing Apple Vision Pro in the operating room for presurgical planning

The Apple Vision Pro with Hoth Intelligence’s (Philadelphia, PA) proprietary application was used in the surgical planning of this case. The Apple Vision Pro is a tethered video-passthrough mixed reality headset. This means that the headset displays the user's real-world view through a recording of the environment that is instantly displayed to the user's eyes through the headset (Figure 2). The system displays the patient’s anatomy in the user’s real-world field of view and enables the user to move around and interact with the 3D model. The software allows for individual manipulation of each anatomic layer. For example, a user may plan to increase the skull and brain transparency in order to visualize the ventricles more clearly.

Qualitative survey

For each clinician-neurosurgery attending, neurosurgery residents who used the technology for presurgical planning completed a usability survey. A general survey developed by the authors was used. The survey questions were Likert scales describing their initial experience and impression of the technology. All participants had never used or worn the Apple Vision Pro before.

Results

**Figure 3:**
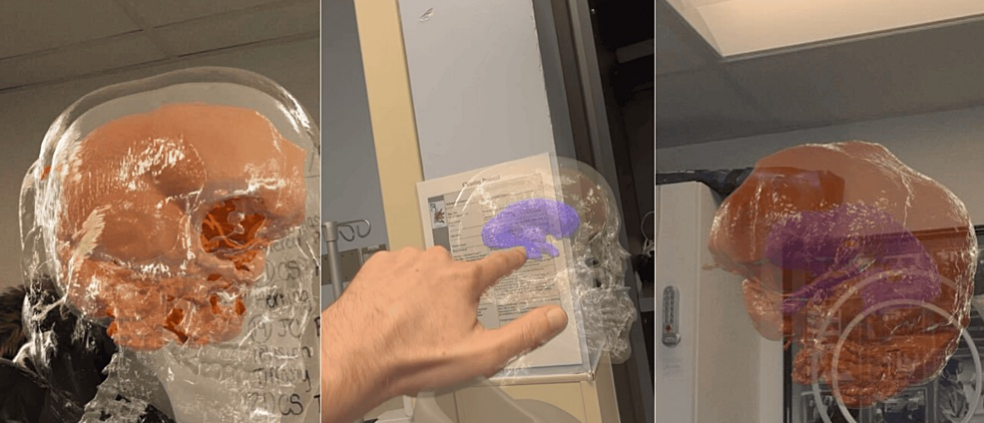
Surgeon's view through the headset 3D model viewed through the Apple Vision Pro showing the various anatomic layers: skull (clear), brain (orange), and ventricles (blue)

Prior to the start of the case, the surgeon used the headset in the operating room to visualize the patient's anatomy. The Apple Vision Pro allowed the surgeon to move around the 3D model and visualize the anatomy from different perspectives. In particular, visualizing the size and location of the ventricles in relation to the brain and skull assisted in planning the approach and entry point (Figure 3, Video 1). After using the technology, the VP shunt placement was performed.

**Video 1:** User's view through the Apple Vision Pro

Given the novelty of this new technology, there is currently no data on the usability or perceived value of the Apple Vision Pro for neurosurgical care. As early users of the technology for visualizing patient-specific 3D anatomy, healthcare providers involved in this case were surveyed about their experience (Table 1). In particular, the participants were surveyed on the perceived quality of the visual display and the comfort of the headset. Overall, surgeons felt that the quality of the 3D anatomy was high and that the visuals felt realistic (4.5/5). The surgeon's view of their real-world surroundings was perceived overall as natural (4.3/5). Despite the headset comfort receiving a score of 3.7/5, the system did not induce cybersickness or eye fatigue, according to the clinicians (4.5/5). Lastly, clinicians rated 4/5 regarding their interest in continuing to use the headset in the operating room.

**Table 1:** Survey responses from clinicians using the Apple Vision Pro

Survey question	Response (n=5)		Average Likert score
Have you used the Apple Vision Pro before?	Yes: 0%		
Did the 3D anatomy feel realistic through the headset? (1-5)	1 - Not at all	5 - Yes very much	4.5
Did your display of the “real world view” feel natural? (1-5)	1 - Not at all	5 - Yes very much	4.3
Was the headset comfortable? (1-5)	1 - Very uncomfortable	5 - Very comfortable	3.7
Did the headset cause any strain on your eyes? (1-5)	1 - A lot of strain	5 - No strain at all	4.5
Would you continue to use this headset in the operating room? (1-5)	1 - Never again	5 - All the time	4

## Discussion

The integration of cutting-edge technologies into the realm of healthcare has witnessed a paradigm shift in the way medical professionals approach diagnostics and surgeries. For the past few years, mixed reality has rapidly entered and expanded within the field of neurosurgery for various purposes, including presurgical planning and intraoperative navigation. Despite the growth of these technologies, the mixed reality headsets used for clinical purposes have largely been unchanged, with clinical use predominantly involving headsets such as the Microsoft Hololens and Magic Leap [3,9-12]. While these are impressive devices, improvements in spatial computing and 3D graphics have enabled major advancements in these technologies. The Apple Vision Pro, which was released in February 2024, is the latest mixed-reality computing device commercially available. Due to its nascency, there is little to no assessment of its clinical and surgical potential. As with all of these mixed reality technologies that preceded the Apple Vision Pro, this headset must be subjected to careful evaluation of its advantages and shortcomings before it is to be readily adopted into the field. Here we present a clinical case report and possibly one of the earliest clinical uses of the Apple Vision Pro for presurgical planning during a shunt revision case.

For this case, the high-quality imaging detail afforded by the Apple Vision Pro enabled the surgeon to visualize the size and shape of the cranial anatomy prior to performing the case. We have used other headsets, such as the Microsoft Hololens 2 (Microsoft Corporation, Washington, USA), for similar types of cases. A notable advantage offered by the Apple Vision Pro over some of the other mixed reality headsets is its improvement in imaging quality. In this case, we were able to clearly appreciate the shape and dimensions of the ventricles and their relative positioning within the brain and skull. Additionally, compared with other headsets that display 3D models as "holograms," the 3D models displayed by the Vision Pro have realistic textures to them, making it feel as if there is a physical model in the room. Due to its greater computing power, the headset allows for a seamless display of high-quality, detailed anatomy renderings of patient anatomy without any drop in performance. This was substantiated by the surgeon surveys, whereby the clinicians scored a 4.5/5 when asked how realistic the models felt. One concern about the Apple Vision Pro was whether the video passthrough display would be disorienting to users. Video passthrough is different from other mixed reality devices, such as the Hololens, in that the user's real-world field of view is recorded and instantly displayed back to them. Issues with other video passthrough devices have been eye strain or cyber sickness. We were encouraged to see that participants responded 4.3/5 when asked how realistic their “real world” view felt and 4.3/5 when asked about eye strain (suggesting little to no strain). It will be important to evaluate the use of the headsets for longer durations to assess whether long-term use introduces any eye fatigue or discomfort. One area that received mixed reviews was the headset’s comfort, receiving a score of 3.7/5. A possible explanation for this feedback is that the headset comes in several different sizes tailored to the user's head size and shape. For this case, the clinicians all used a single headset, and, thus, for several participants, the headset fit was likely suboptimal.

The release of the Apple Vision Pro represents a new era of mixed reality for the medical community. Superior computing power, various cameras, and high-resolution sensors create a mixed-reality experience that surpasses many of the devices that preceded it. This report describes a limited use of the technology in surgical planning; however, we felt it was imperative to describe the early use of the device given its recent emergence in the market. Despite the excitement behind this system, it is critical that the technology be carefully scrutinized in order to improve upon it as well as find the right role for it in the field of medicine. A key question about the headset will be whether surgeons can and are willing to use it intraoperatively and potentially for neuronavigation. Based on our limited understanding of the headset's sensor technology, it is likely that the spatial computing and precision will exceed even those of traditional navigation. That is, the Apple Vision Pro should have the capability to register the 3D models onto a patient and track instruments with comparable accuracy to image guidance systems currently used in neurosurgery. However, concerns over headset ergonomics (i.e., fit and weight) and potential eye fatigue may limit this type of use. There are many opportunities that should be explored in future studies, such as the display of endoscopic or microscopic video feeds through the headset, the visualization of medical scans for diagnostic purposes, and the potential for intraoperative navigation. While this report describes an early use of the headset, we anticipate the rapid emergence of this technology in the field of neurosurgery. Here we found that the headset, despite its recent release, can be used nicely for surgical planning and visualization. The visual display and processing power of the system are unparalleled, giving the users a unique display of patient 3D anatomy. Therefore, it is our hope that this work prompts further work and investigation with the device across the neurosurgical community.

## Conclusions

As mixed reality continues to expand in the field of neurosurgery, the release of the Apple Vision Pro represents a major technological milestone. Based on this simple case report, the headset seems appropriate for presurgical planning and anatomical visualization. This new mixed-reality device will enable features and functionalities previously unavailable on earlier headsets. Despite the promise of this technology, it is imperative and expected that the device be rigorously assessed in both preclinical and clinical studies in order to improve upon the technology and further refine the role it can play in patient management.
